# Phylogenetic analysis and in-depth characterization of functionally and structurally diverse CE5 cutinases

**DOI:** 10.1016/j.jbc.2021.101302

**Published:** 2021-10-13

**Authors:** Vera Novy, Leonor Vieira Carneiro, Jae Ho Shin, Johan Larsbrink, Lisbeth Olsson

**Affiliations:** 1 Division of Industrial Biotechnology, Department of Biology and Biological Engineering, Chalmers University of Technology, Gothenburg, Sweden; 2 Wallenberg Wood Science Center, Chalmers University of Technology, Gothenburg, Sweden

**Keywords:** CE5, cutinases, biodiversity, phylogenetic analysis, docking analysis, enzyme kinetics, structure–function, suberin, cutin, plastic degradation, CE5, carbohydrate esterase family 5

## Abstract

Cutinases are esterases that release fatty acids from the apoplastic layer in plants. As they accept bulky and hydrophobic substrates, cutinases could be used in many applications, ranging from valorization of bark-rich side streams to plastic recycling. Advancement of these applications, however, requires deeper knowledge of cutinases’ biodiversity and structure–function relationships. Here, we mined over 3000 members from carbohydrate esterase family 5 for putative cutinases and condensed it to 151 genes from known or putative lignocellulose-targeting organisms. The 151 genes were subjected to a phylogenetic analysis, which showed that cutinases with available crystal structures were phylogenetically closely related. We then selected nine phylogenic diverse cutinases for recombinant production and characterized their kinetic activity against para-nitrophenol substrates esterified with consecutively longer alkyl chains (*p*NP-C_2_ to C_16_). Each investigated cutinase had a unique activity fingerprint against the tested *p*NP substrates. The five enzymes with the highest activity on *p*NP-C_12_ and C_16_, indicative of activity on bulky hydrophobic compounds, were selected for in-depth kinetic and structure–function analysis. All five enzymes showed a decrease in *k_cat_* values with increasing substrate chain length, whereas *K_M_* values and binding energies (calculated from in silico docking analysis) improved. Two cutinases from *Fusarium solani* and *Cryptococcus* sp. exhibited outstandingly low *K_M_* values, resulting in high catalytic efficiencies toward *p*NP-C_16_. Docking analysis suggested that different clades of the phylogenetic tree may harbor enzymes with different modes of substrate interaction, involving a solvent-exposed catalytic triad, a lipase-like lid, or a clamshell-like active site possibly formed by flexible loops.

Cutinases (EC 3.1.1.74) are small serine esterases (20–25 kDa) that show hydrolytic, esterification, and transesterification activity on hydrophobic compounds (1). They belong to the α/β hydrolase superfamily, and similar to lipases they possess a Ser–His–Asp catalytic triad and an oxyanion hole for transition state stabilization of the substrate. Unlike lipases, cutinases have the catalytic triad located in a shallow binding cleft, exposed to the solvent and thus can act on hydrophobic, high-molecular-weight molecules without the need for surface activation (1, 2).

The native substrates of cutinases are cutin and suberin (1, 2), which represent the main components of the apoplastic barrier in higher plants (3). These polyesters are made from long fatty acid chains, C_16_-C_18_ for cutin and C_16_-C_34_ for suberin, which form links to each other and to a polyaromatic, lignin-like domain, *via* glycerol or ferulic acid head groups (4, 5). Together, these polymers create a complex three-dimensional network with important functions for the plant such as management of water-, solute-, and gas translocation as well as providing physical strength and resistance to pathogenic attack (6). The cutin- or suberin-derived fatty acids contain a diverse combination of functional groups, including epoxy-, alcohol-, and carboxy groups as well as unsaturated bonds (3). These traits make plant-cuticle derived fatty acids interesting polymer precursors for fine chemical applications that have the potential to replace conventional, petrochemical compounds (7).

Previous studies have suggested that cutinases are secreted by plant pathogens to either disrupt the protective apoplastic layer to allow access to the underlying cells and cell walls or, alternatively, to enable direct use of the released fatty acids as carbon sources (6, 8). Industrial use of cutinases includes exploiting their esterification and transesterification activity (2) to synthesize new compounds, such as flavor esters (9). More significant, however, is their ability to hydrolyze ester bonds of bulky and/or hydrophobic polymers. Their ability to act on man-made polymers, such as the aromatic plastic PET (polyethylene terephthalate) or the aliphatic plastic PLA (polylactic acid), holds promise to make cutinases key tools in plastic degradation, recycling, and upgrading (1, 9, 10). Moreover, cutinases could be applied on their natural substrates to release cutin- and suberin-derived fatty acids from plant cuticles, such as the bark and root of trees. Releasing such fatty acids could add a high-value product to conventional forest-based biorefinery processes, facilitating their economic feasibility.

The biodiversity of organisms encoding cutinases is vast. Within the Carbohydrate-Active Enzymes database (CAZy, www.cazy.org, (11)), cutinases are found in Carbohydrate Esterase family 5 (CE5), which today contains over 3000 entries. Enzymes have been described to originate chiefly from bacteria and eukaryotes (mainly fungi), with candidates of both origins being able to act on cutin, suberin, or both (1, 2, 9). The CE5 family additionally contains enzymes with acetyl xylan esterase activity (EC 3.1.1.72). Cutinases not enlisted in the CE5 family have also been described, where dedicated PETases, *e.g.*, from *Ideonella sakaiensis*, are the most prominent ones (12). Here, we solely focused on cutinases that are active on plant biomass, belonging to the CE5 family.

The sequence identity between cutinases has been reported to be quite low (1, 13), and extensive functional development based on evolution has been described for this type of enzyme (13). The corresponding broad diversity in structure–function relationships could be exploited to identify suitable cutinase candidates for a specific application (10). However, previous studies only looked at a few sequences (1, 13), and in-depth information on biodiversity, enzyme kinetics, and enzyme structure–function relationships within CE5 is lacking, hindering the full exploration of cutinases. As of July 2021, only nine solved crystal structures of CE5 cutinases have been reported, and as we demonstrate in this study, most of them are phylogenetically close. Further, using the most common model substrate, *para*-nitrophenol (*p*NP) esterified with fatty acids of increasing chain length (*p*NP-C_n_) (1), most studies only state the volumetric activity of selected cutinases (14, 15, 16, 17, 18, 19, 20, 21). Reports of the kinetic parameters *K*_*M*_ (indicating substrate affinity), *k*_*cat*_ (maximal turnover rate), *k*_*cat*_*/K*_*M*_ (specificity constant), or quantified enzyme–ligand interactions are much rarer (22, 23). Such detailed information is, however, vital to understand the structure–function relationship of cutinases and hence brings these promising types of enzymes closer to application.

In the present study, we explore the biodiversity of CE5 cutinases and provide in-depth information on enzyme kinetics and structure–function relationships of phylogenetically, structurally, and kinetically diverse cutinases. As CE5 is a polyspecific family, we filtered its members to focus on ∼150 putatively active cutinases, which were phylogenetically analyzed. Out of these, nine cutinases from across the phylogenetic tree were selected and recombinantly expressed in *Pichia pastoris*, and the differences in sequence were reflected in their individual activities toward *p*NP-C_2_ to *p*NP-C_16_ substrates as well as in their modeled protein structures. The five candidates with the highest activity on *p*NP-C_16_, indicating activity on bulky and hydrophobic substrates, were subjected to in-depth kinetic characterization and structure–function analysis by *in silico* docking analyzes. Our results shed light on the diversity of CE5 cutinases and have implications for both their utilization and future enzyme discovery efforts.

## Results and discussion

### Phylogenetic analysis of CE5

By 2021, over 3000 members are listed in the CAZy database CE5 family. We sorted the listed enzymes in CE5 to condense the family to putative cutinases and subjected the resulting entries to a phylogenetic analysis. Nine enzymes (summarized in Table 1), spanning the phylogenetic tree, were selected for cloning and characterization against *p*NP-substrate with increasing chain lengths.

**Table 1:** Description of cutinases selected for recombinant production and kinetic characterization

Protein id	Uniprot accession	Source organism	Reference
*Cc*Cut	B9U443	*Coprinopsis cinerea* Fungi; Basidiomycete; Filamentous	(18)
*Sc*Sub	A0A5P3LVN0	*Streptomyces scabiei*; Bacteria; Actinobacteria	(20)
*An*Cut	Q5B2C1	*Aspergillus nidulans* Fungi; Ascomycete; Filamentous	(8)
*Pb*Cut	Q9Y7G8	*Pyrenopeziza brassicae* Fungi; Ascomycete; Filamentous	(44)
*Sc*Cut	S4VCH4	*Sirococcus conigenus* Fungi; Ascomycete; Filamentous	(19)
*Mc*Cut	A1DGN0.1	*Aspergillus fischeri*^a^ Fungi; Ascomycete; Filamentous	(45)
*Tt*Cut	G2RAE6	*Thermothielavioides terrestris* Fungi; Ascomycete; Filamentous	(15)
*Fs*Cut	P00590.1	*Fusarium solani* Fungi; Ascomycete; Filamentous	(46)
*Csp*Cut	Q874E9	*Cryptococcus* sp Fungi; Basidiomycete; Yeast	(47)

a Gene detected in *Malbranchea cinnamomea* transcriptome in in-house study, annotated as *Aspergillus nidulans* cutinase A1DGN0.1.

A scheme was devised to sort CE5 sequences into putative cutinases according to the decision tree depicted in Figure S1. For this, entries were first evaluated toward their likelihood to result in a mature, active enzyme. In a second step, the correlation of either the enzyme or the encoding organism to lignocellulolytic activity was assessed (*e.g.*, plant pathogens), indicating potential suberin- or cutin-degrading activity. The resulting list of genes contained 151 putative cutinases with 41 from bacterial and 110 from fungal organisms, suggesting a predominance of cutinases in fungal lignocellulose degraders. Of the 41 bacterial cutinases, 32 are from the *Mycobacterium* genus within the Actinobacteria phylum, where species of *Mycobacterium* spp (16 species) and *Mycolicibacter* spp (14 species) were most common. The fungal cutinases originated from a broader set of organisms, where ascomycetes were more common than basidiomycetes. Here, the most represented genera are *Aspergillus* (12 species), *Fusarium* (26 species), and *Pyricularia* (15 species).

**Figure 1:**
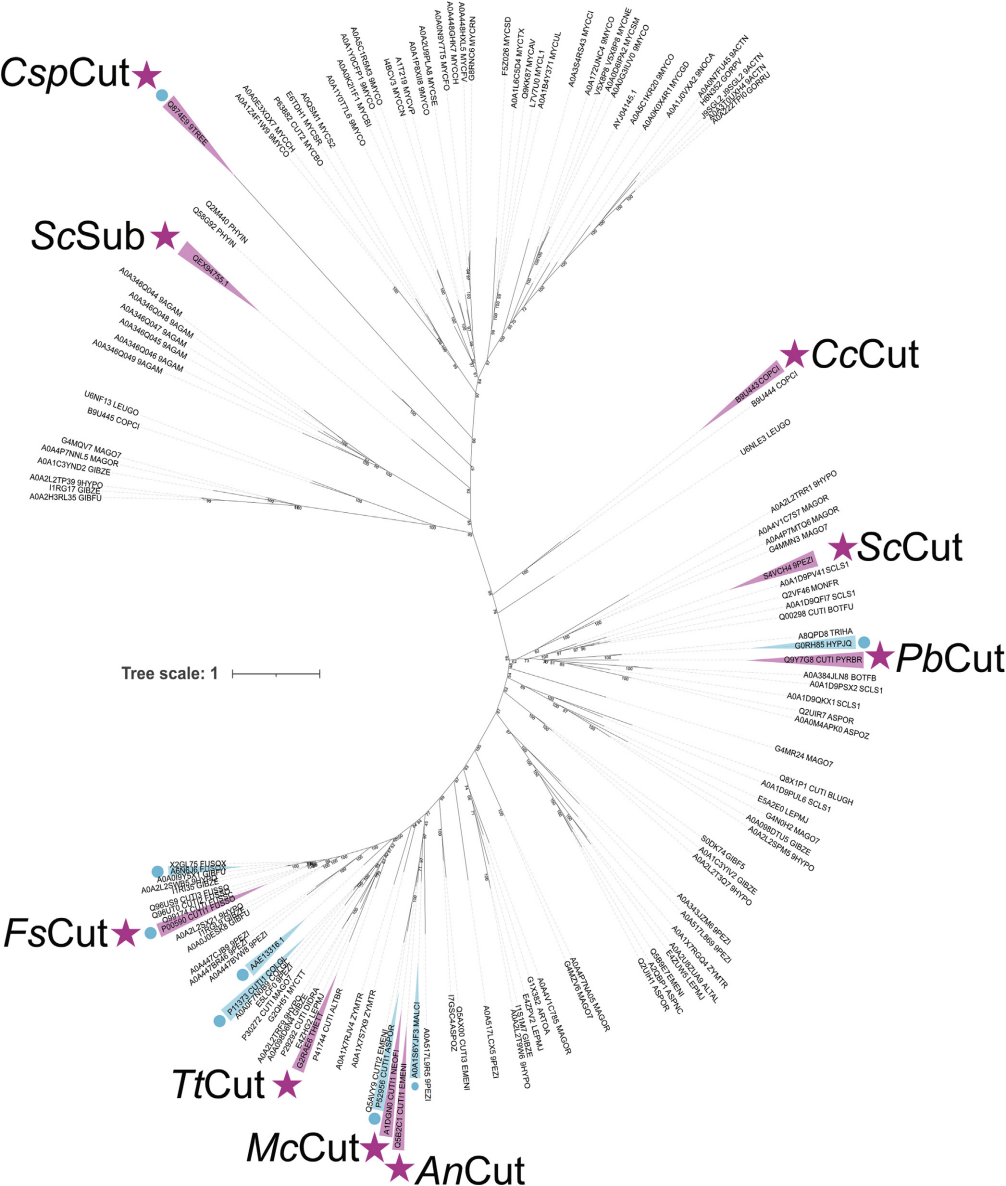
**Phylogenetic tree of CE5 cutinases.** Proteins chosen for cloning and characterization are marked in *magenta* and *stars* with the abbreviation used in this study (Table 1). Proteins with solved crystal structures are highlighted in turquois and additionally marked with a *circle* for clarity. A decision tree on the narrowing down process of the CE5 entries is summarized in Figure S1. The main organisms found in each clade are summarized in Figure S2. A summary of all entries is submitted with this paper (supporting information).

The phylogenetic tree constructed from the 151 cutinase sequences is displayed in Figure 1. The tree revealed nine main clades, or clusters, of which a detailed description is given in Figure S2 (1). The amino acid sequence identities between the 151 cutinases analyzed in this study ranged from 8 to 93% (as analyzed with Clustal Omega at https://www.ebi.ac.uk/), proving the vast sequence diversity of this enzyme family. Our results are in line with a previous study (13), where analysis of the evolutionary history of cutinases from five ascomycetes revealed “extreme sequence diversity.” Transcript profiling for categorizing regulatory patterns has further suggested that phylogenetically related cutinase genes in *Magnaporthe grisea* underwent extensive sub- and neo-functionalization (13). This implies that the genome of a single organism can harbor and differentially express cutinases with low sequence identity and broad functionality, which was likely a chief driver for the niche differentiation of some fungi into plant parasites (6, 8, 13).

Supporting the proposed physiological function of cutinases, the majority of the fungal cutinases selected in this study are from plant pathogens (Fig. S2). The function of cutinases from the human and animal pathogen *Mycobacterium* as esterases, in contrast, is likely parallel to that of other enzymes accepting hydrophobic substrates and might contribute to the pathogenicity by degrading the cell membrane of the infected host. Because they lack cutin-degrading activity (24), they were placed outside the scope of this study. Our phylogenetic analysis showed that the *Mycobacterium* cutinases form their own discrete clade clearly apart from enzymes from other clades, which mainly harbor fungal enzymes (Fig. S2, “clade G”).

The selected 151 sequences encompass nine solved cutinase crystal structures (Fig. 1), six of which are in one area of the phylogenetic tree (Fig. S2, clades A, B, and C). From the analyzed cutinases, the one from *Fusarium solani* (accession number P00590) is the most studied with 44 resolved structures of the wild-type enzyme and various variants.

From the phylogenetic tree, the next step was to identify cutinases from different organisms for recombinant production in *P. pastoris*, followed by kinetic characterization. Cutinases from diverse sources have been successfully produced in *P. pastoris*, suggesting it to be a suitable host for these enzymes (14, 15, 17, 19, 22, 25). The nine resulting enzyme candidates are listed in Table 1, which also summarizes the herein used abbreviations, accession numbers, and source organisms. The choice of target genes for recombinant production was based on several criteria. From the phylogenetic analysis, at least one candidate of each phylum (*i.e.*, basidiomycete, ascomycete, and actinobacterium) and main observed genera (*Fusarium*, *Aspergillus*) should be represented. Secondly, the biodiversity should be spanned, using both enzymes from the well-analyzed clades (Fig. S2, clades A, B, and C) as well as from underinvestigated areas of the tree (Fig. 1). Thirdly, from our previous transcriptome studies of *Malbranchea cinnamomea* and *Thielavia terrestris* (26, 27), we have identified two cutinases significantly overexpressed on lignocellulolytic substrates, which were also added to the list. Finally, we added a cutinase from *Sirococcus conginenus* because of its suggested acid tolerance (19), a trait important in a biorefinery context where pretreatment results in low pH of the biomass slurries (25).

### Kinetic and structural characterization of nine phylogenetically diverse cutinases

**Figure 2:**
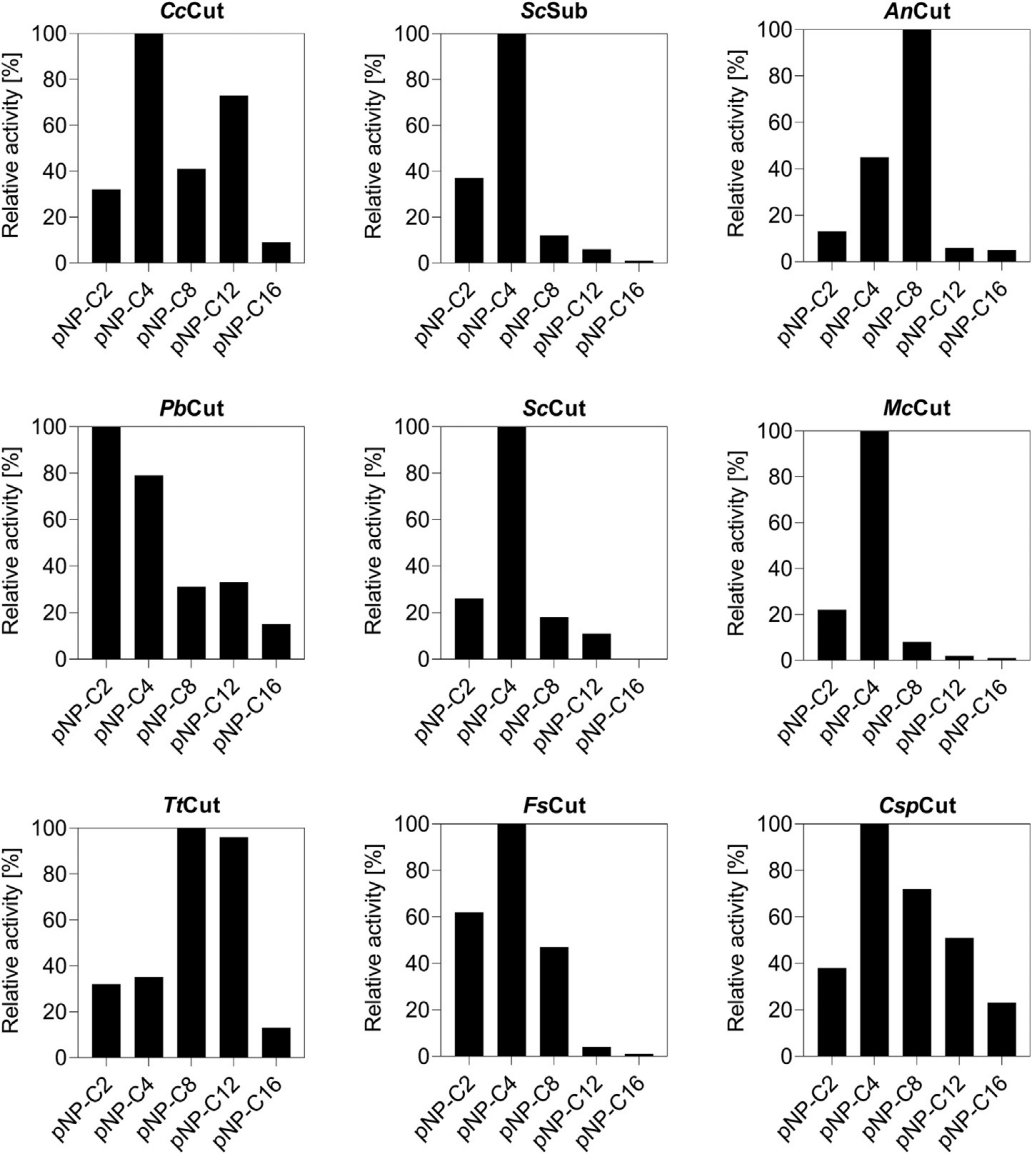
**Activity profile of the nine selected cutinases against *p*NP-substrates determined at pH 7.** Depicted is the relative specific activity (normalized on the highest activity measured for each enzyme) measured in the concentrated supernatant of *P. pastoris* cultivations. Values are averages from duplicate experiments. A summary of the mean specific activities (in U mg^−1^ total protein in the cultivation supernatant) and the data spread can be found in Table S1.

The “activity fingerprints” (*i.e.*, the relative specific activity of each enzyme against *p*NP-C_2_ to *p*NP-C_16_) were determined (Fig. 2), and a summary of the mean specific activities (in U mg^−1^ total protein in the cultivation supernatant) and the spread can be found in Table S1. Each cutinase showed a unique fingerprint. The activity maximum (“100%” activity, Fig. 2) was reached on shorter chain substrates: *p*NP-C_2_ (*Pb*Cut), *p*NP-C_4_ (*Cc*Cut, *Sc*Sub, *Sc*Cut, *Mc*Cut, *Fs*Cut, and *Csp*Cut), or *p*NP-C_8_ (*An*Cut, *Tt*Cut). While enzymes with activity maxima at *p*NP-C_2_ to C_6_ seem to be common (14, 15, 16, 17, 18, 19, 20, 21), the preference of *An*Cut and *Tt*Cut for *p*NP-C_8_ appears to be less common as only a few cutinases have been described to prefer these longer chain fatty acid esters (22).

With the longest chain length substrates, *p*NP-C_12_ and *p*NP-C_16_, all nine cutinases had lower activity than on the short substrates (Fig. 2), an effect also observed in other studies (15, 16, 17, 18, 19, 20, 21, 22). The extent of the reduced activity was, however, individual for each enzyme (Fig. 2), and the reduction is likely a result of the increased hydrophobicity caused by the long, hydrophobic alkyl chains of these substrates (28). In addition, the enzyme must be able to accommodate the long and bulky substrate in the active site and binding cleft, for which some cutinases might have a more suitable surface topology than others.

To elucidate the underlying reason for the divergent performance of the nine cutinases, we looked at their overall and active site structure, either using solved crystal structures (*Fs*Cut, *Csp*Cut) or by creating enzyme models, using three modeling algorithms (Phyre2, iTasser, SWISS-MODEL). After extensive examination of the models and comparison with their templates (*i.e.*, alignment of the catalytic triad and the oxyanion hole, formation of disulfide bridges, configuration of N- and C-termini), one model for each enzyme was chosen for further analysis. An overview of the results is given in Table 2, the structures are displayed in Figure S3.

**Table 2:** Structural overview of the nine selected cutinases

Protein id	Catalytic triad/oxyanion hole	Hydrophobic area	Catalytic triad location
*Cc*Cut	Ser116, His180, Asp168/Thr42, Gln117	Around binding cleft	Shallow binding cleft, exposed to solvent
*Sc*Sub	Ser87, His168, Asp155/Thr15, Gln88, Ser54	All binding face	Deep binding cleft, exposed to solvent
*An*Cut	Ser127, His195, Asp182/Ser49, Gln125	Around binding cleft	Embedded deeper in the enzyme
*Pb*Cut	Ser118, His183, His170/Thr43, Gln119	All binding face	Deep narrow binding cleft, proposed involvement of loop
*Sc*Cut	Ser118, His182, Asp169/Thr42, Gln119	Two loops flanking binding cleft	Embedded deeper in the enzyme
*Mc*Cut	Ser125, His193, Asp180/Ser47, Gln126	Two loops flanking binding cleft	Shallow binding cleft, exposed to solvent
*Tt*Cut	Ser136, His204, Asp191/Ser59, Gln137	Around binding cleft	Encased in a tunnel
*Csp*Cut	Ser85, His179, Asp165/Thr17, Gln86	Two loops flanking binding cleft	Shallow binding cleft, exposed to solvent
*Fs*Cut	Ser120, His188, Asp175/Ser42, Gln121, Asn84	Two loops flanking binding cleft	Shallow binding cleft, exposed to solvent

Surface models and active side configurations of each enzyme are shown in Figure S3.

Except for *Pb*Cut, all enzymes contain a Ser-His-Asp catalytic triad, typical for cutinases. *Pb*Cut (Table 2; Fig. S3) harbors a Ser-His-His triad. Although less common, this unconventional catalytic triad has been described for some serine proteases (29). Mutational studies (30) have shown, however, that removal of the Asp-replacing His only affects the activity marginally, suggesting that *Pb*Cut likely harbors a functional dyad.

The oxyanion hole is also conserved across the investigated cutinases and is composed of a Thr or a Ser and a Gln residue, with both the backbone amides and/or the amino acid side chains being involved (Table 2; Fig. S3).

Despite the similarity of the catalytic triad, the enzymes' architecture around the binding cleft can vary significantly (Table 2; Fig. S3). In the cases of *Cc*Cut, *Mc*Cut, *Csp*Cut, and *Fs*Cut, the catalytic triad and oxyanion hole are located in a shallow cleft with the amino acid residues of the catalytic triad exposed to the solvent. This has been described as a unique feature of cutinases (31), setting them apart from lipases, which have a lid covering the active site. In contact with the hydrophobic substrate, the lid is removed and the catalytic triad exposed to the substrate (“surface/interfacial activation”) (32). Interestingly, we found that in some cutinases the catalytic triad is embedded in a tunnel (*Tt*Cut) or deeper within the enzyme (*An*Cut, *Sc*Cut). In literature, one cutinase (from *Trichoderma reesei*, accession number G0RH85) has been described where an N-terminal loop with high flexibility is covering the active site cleft (33). In that study, it was hypothesized that this loop has a function similar to the lid observed in lipases and plays an active part in substrate recognition and binding (33). This would place this and similar cutinases between lipases and cutinases as traditionally described. In this study, the *T. reesei* cutinase crystal structure G0RH85 was the main template for four cutinases, with *Cc*Cut, *Sc*Sub, and *Sc*Cut being modeled after the enzyme in its native form (PDB: 4PSC or 4PSD), where the lid is “closed.” Only *Pb*Cut was modeled after the complexed form (C11Y4 phosphonate inhibitor; PDB: 4PSE), where the lid is swung out. The proposed function of the lid is discussed later in the paper, when analyzing the structure–function relationship of *Cc*Cut and *Pb*Cut in more detail. It is worth mentioning that all four sequences (*Cc*Cut, *Sc*Sub, *Sc*Cut, and *Pb*Cut) are located in the less investigated parts of the phylogenetic tree (Fig. S2), where *Cc*Cut, *Sc*Cut, and *Pb*Cut are very close (in or close to clade F; Fig. S2).

**Figure 3:**
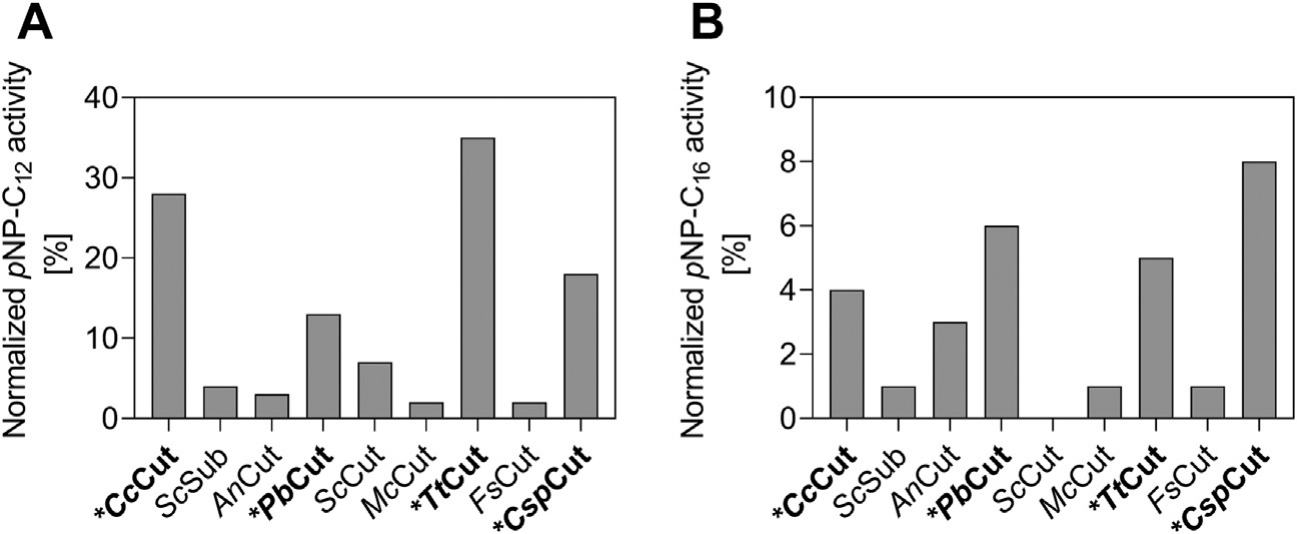
**Identifying cutinases for in-depth kinetic analysis.** Depicted is the normalized *p*NP-C_12_ (*A*) and *p*NP-C_16_ (*B*) activity of all nine cutinases, given as percentage of the sum of all *p*NP (C_2_ to C_16_) activities measured for each enzyme. Data were extracted from Figure 2. Values are averages from duplicate experiments. A summary of the mean specific activities (in U mg^−1^ total protein in the cultivation supernatant) and the data spread can be found in Table S1. *y*-axes adjusted for clarity.

One active site configuration that differed from the other architectures is that of *Sc*Sub (Fig. S3). This enzyme was the only CE5 member denoted “suberinase” (20) and has a much wider and deeper active site cleft. Considering the complex ultrastructure of suberin consisting of longer chain fatty acids than cutin (3, 4), *Sc*Sub might be a result of evolutionary functionalization toward suberin as substrate. Although *Sc*Sub did not show above average activity against *p*NP-C_12_ and *p*NP-C_16_ in this study (Figs. 2 and 3), *Sc*Sub still might be an excellent candidate for suberin degradation.

Aside from the configuration of the active site, the enzymes differ in the amount and distribution of hydrophobic patches on the enzyme surface. As cutinases' natural substrate is highly hydrophobic, this trait is pivotal in facilitating efficient substrate recognition (1), and all cutinases analyzed here have hydrophobic areas around the proposed binding clefts (Fig. S3). However, there are differences in the distribution of these hydrophobic areas, as highlighted in Figure S3 and summarized in Table 2. The hydrophobic areas can either be limited to a comparatively narrow space around the active site cleft (*An*Cut), extend over the enzyme surface forming a hydrophobic face (*Sc*Sub, *Pb*Cut, *Tt*Cut), or be found on two loops flanking the binding cleft (“left and right” of the binding cleft in Fig. S3; *Sc*Cut, *Mc*Cut, *Csp*Cut, *Fs*Cut). These differences might suggest different modes of action toward the substrate or indicate differences in substrate preferences.

### Detailed kinetic characterization and structure–function analysis of cutinases with high *p*NP-C_12_ and *p*NP-C_16_ activity

From the initial activity fingerprint of the nine cutinases (Fig. 2), *Cc*Cut, *Pb*Cut, *Tt*Cut, and *Csp*Cut stood out for their superior *p*NP-C_12_ and *p*NP-C_16_ activity (Fig. 3). These enzymes have extensive hydrophobic areas flanking the binding cleft (Table 2; Fig. S3). *Cc*Cut, *Pb*Cut, and *Csp*Cut further harbor the open binding cleft, with the active site exposed to the solvent. Although such an active site topology has been shown to aid activity on long chain substrates (23, 34), it does not appear to be a decisive feature as comparison of *Sc*Sub (wide and open binding cleft; low *p*NP-C_12_ and C_16_ activity) and *Tt*Cut (tunnel; high *p*NP-C_12_ and C_16_ activity) shows (*cf.*, Table 2; Figs. 3 and S3). Apart from *Cc*Cut, *Pb*Cut, *Tt*Cut, and *Csp*Cut, we also included *Fs*Cut in the kinetic characterization, as it is the most well-studied cutinase and provides a good basis for comparison. Activities for *Fs*Cut were found to be comparable to previously reported data (21).

In previous studies, cutinases have mainly been characterized by analyzing volumetric activities towards various *p*NP-substrates (14, 15, 16, 17, 18, 19, 20, 21), while in-depth characterization to determine kinetic parameters is much rarer (22, 23). Here we performed an in-depth characterization to determine the kinetic parameters of *Cc*Cut, *Pb*Cut, *Tt*Cut, *Csp*Cut, and *Fs*Cut against *p*NP-C_2_ to *p*NP-C_16_ (Table 3).

**Table 3:** In-depth kinetic^a^ and docking^b^ analysis of five cutinases

	*Cc*Cut	*Pb*Cut	*Tt*Cut	*Fs*Cut	*Csp*Cut
*K*_*M*_ (mM)					
*p*NP-C_2_	34.31 ± 0.62	11.73 ± 0.15	24.81 ± 3.43	7.31 ± 0.25	9.54 ± 0.99
*p*NP-C_4_	3.56 ± 0.15	1.71 ± 0.17	7.08 ± 0.09	1.67 ± 0.04	5.54 ± 0.50
*p*NP-C_8_	1.67 ± 0.18	0.54 ± 0.07	0.39 ± 0.01	0.72 ± 0.01	0.05 ± 0.00
*p*NP-C_12_	2.76 ± 0.05	0.08 ± 0.01	0.21 ± 0.00	0.05 ± 0.01	0.05 ± 0.00
*p*NP-C_16_	2.90 ± 0.20	0.04 ± 0.01	0.50 ± 0.09	0.01 ± 0.00	0.05 ± 0.00
*k*_*cat*_ (s^−1^)					
*p*NP-C_2_	41.37 ± 1.46	11.84 ± 0.40	65.79 ± 7.96	29.96 ± 1.16	29.74 ± 1.80
*p*NP-C_4_	8.96 ± 0.39	6.45 ± 0.52	15.92 ± 0.06	7.70 ± 0.19	13.52 ± 0.25
*p*NP-C_8_	1.70 ± 0.17	1.43 ± 0.09	2.30 ± 0.06	6.69 ± 0.24	3.81 ± 0.27
*p*NP-C_12_	0.22 ± 0.01	0.20 ± 0.00	0.07 ± 0.00	1.22 ± 0.03	3.49 ± 0.06
*p*NP-C_16_	0.06 ± 0.00	0.08 ± 0.00	0.09 ± 0.01	0.15 ± 0.00	3.38 ± 0.12
*k*_*cat*_/*K*_*M*_ (mM^−1^ s^−1^)					
*p*NP-C_2_	1.21 ± 0.02	1.01 ± 0.02	2.66 ± 0.05	4.10 ± 0.02	3.13 ± 0.14
*p*NP-C_4_	2.52 ± 0.00	3.79 ± 0.07	2.25 ± 0.04	5.71 ± 0.08	2.51 ± 0.27
*p*NP-C_8_	1.02 ± 0.01	2.65 ± 0.19	5.81 ± 0.05	9.25 ± 0.26	83.20 ± 4.20
*p*NP-C_12_	0.08 ± 0.00	2.50 ± 0.18	0.31 ± 0.01	22.78 ± 2.09	65.97 ± 0.60
*p*NP-C_16_	0.02 ± 0.00	2.07 ± 0.25	0.19 ± 0.02	10.60 ± 1.06	70.68 ± 6.47
Binding energy (kcal mol^−1^)^b^					
*p*NP-C_2_	−13.5	−23.8	−22.7	−28.6	−12.5 (−21.7)^c^
*p*NP-C_4_	−11.3	−21.8	−26.6	−36.7	−15.4 (−24.9)^c^
*p*NP-C_8_	−25.7	−30.2	−29.5	−23.9	−19.4 (−30.1)^c^
*p*NP-C_12_	−31.2	−37.7	−33.8	−31.7	−27.0 (−29.7)^c^
*p*NP-C_16_	−32.5	−42.0	−38.3	−31.0	−36.5 (−40.9)^c^

a Cutinases were purified by affinity chromatography. Depicted are mean values and the spread from duplicate experiments.

b From *in silico* docking analyzes. Constraints and docking scores are summarized in Table S2.

c Binding energies in brackets are calculated for the *in silico* Gly176Leu variant.

All five cutinases showed decreased *K*_*M*_ values (indicating improved substrate affinity) with increasing substrate chain length, indicating a better fit in the active site: the *K*_*M*_ values decreased from *p*NP-C_2_ to *p*NP-C_16_ by 12-, 302-, 49-, 198-, and 525-fold for *Cc*Cut, *Pb*Cut, *Tt*Cut, *Csp*Cut, and *Fs*Cut, respectively (Table 3). The lowest *K*_*M*_ values were, however, enzyme-dependent, with *Cc*Cut preferring *p*NP-C_8_, *Tt*Cut *p*NP-C_12_, and *Pb*Cut, *Csp*Cut, and *Fs*Cut *p*NP-C_16_. This shows that all enzymes have an increased affinity toward the more native-like substrates, as *p*NP-C_16_ closely resembles ferulic palmitate, a major building block in both cutin and suberin (35). In kinetic characterizations of *Glomerella cingulata* and *Aspergillus oryzae* cutinases, a similar trend, *i.e.*, increasing substrate affinity with longer chain *p*NP-substrates, has been observed (21, 23).

**Figure 4:**
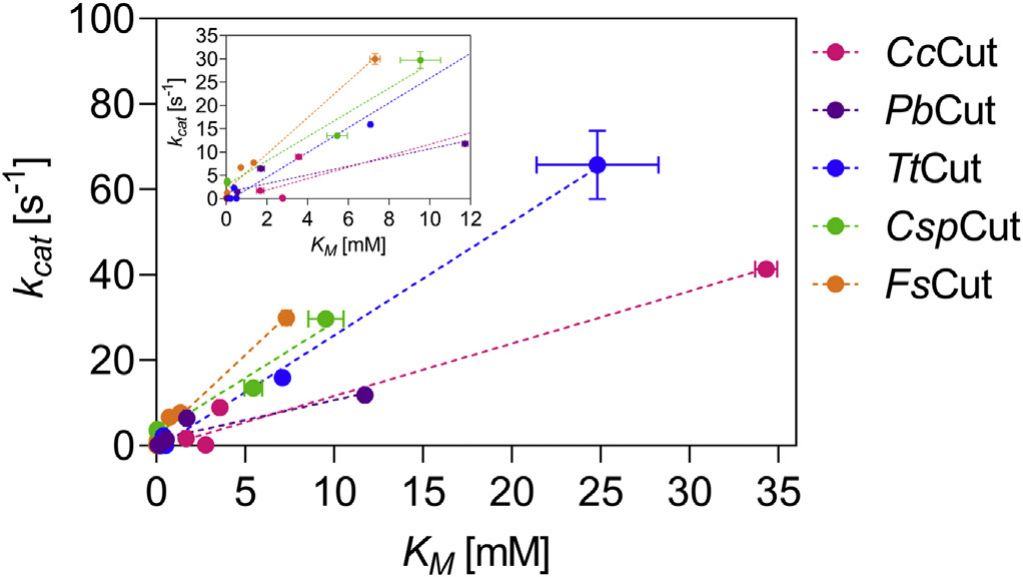
**Correlation between *K***_***M***_**and *k***_***cat***_**in kinetic characterization of *Cc*Cut, *Pb*Cut, *Tt*Cut, *Csp*Cut, and *Fs*Cut using *p*NP-C**_**2**_**to *p*NP-C**_**16**_**as substrates.** A summary of the kinetic parameters can be found in Table 3. The *dotted line* represents the linear regressions of each data set (R^2^ 0.85–0.99). Insert for smaller *K*_*M*_ (0–12 mM) and *k*_*cat*_ (0–35 s^−1^) values was added for clarity.

In contrast to the apparent better fit of longer substrates in the cutinase active sites, as indicated by decreasing *K*_*M*_ values, *k*_*cat*_ decreased for all enzymes with increasing substrate chain length (Table 3). To investigate this correlation between substrate affinity and turnover rate further, *K*_*M*_ was plotted against *k*_*cat*_ for each of the five cutinases assayed against the five *p*NP-substrates. The resulting plots are summarized in Figure 4. In all cases the decreasing *K*_*M*_ correlated well with decreasing *k*_*cat*_ values, resulting in a positive slope of the *K*_*M*_-*k*_*cat*_ plots (Fig. 4). The slope, however, differed, with *Pb*Cut (0.93; R^2^ 0.85) and *Cc*Cut (1.23; R^2^ 0.97) showing a more moderate increase, and *Tt*Cut (2.66; R^2^ 0.99), *Csp*Cut (2.60; R^2^ 0.97), and *Fs*Cut (3.90; R^2^ 0.99) a steeper raise.

Catalytic efficiency is important when choosing an enzyme for a given application. In our study, *Csp*Cut and *Fs*Cut were the only two enzymes, where the very low *K*_*M*_ observed with *p*NP-C_8_ to *p*NP-C_16_ offset losses in *k*_*cat*_, resulting in an improved catalytic efficiency, *i.e.*, *k*_*cat*_/*K*_*M*_, with increasing *p*NP-substrate length (Table 3). A similar trend of *k*_*cat*_ and *K*_*M*_ for long chain *p*NP-substrates has so far only been described for the cutinase from *G. cingulata* (22).

For all enzymes, the binding is improving, *i.e.*, binding energy is decreasing, with increasing substrate chain length (Table 3), and the improved binding is mirrored by the decreasing *K*_*M*_-values (Fig. S4). The calculated *in silico* binding energy can be useful to estimate the substrate affinity in cutinase enzymes. However, differences in the correlations could be detected (Fig. S4), where similar decreases in binding energies resulted in a more (*Tt*Cut, *Pb*Cut, *Csp*Cut) or less (*Cc*Cut, *Fs*Cut) pronounced improvement in ln(*K*_*M*_) (Table 3; Fig. S4). One aspect is that, although high binding energies are pivotal in facilitating efficient enzyme–ligand interaction, strong interaction of the substrate with the active side amino acid residues can result in slow product release, preventing the onset of a new catalytic cycle (36).

### The five cutinases show different modes of substrate interaction

**Figure 5:**
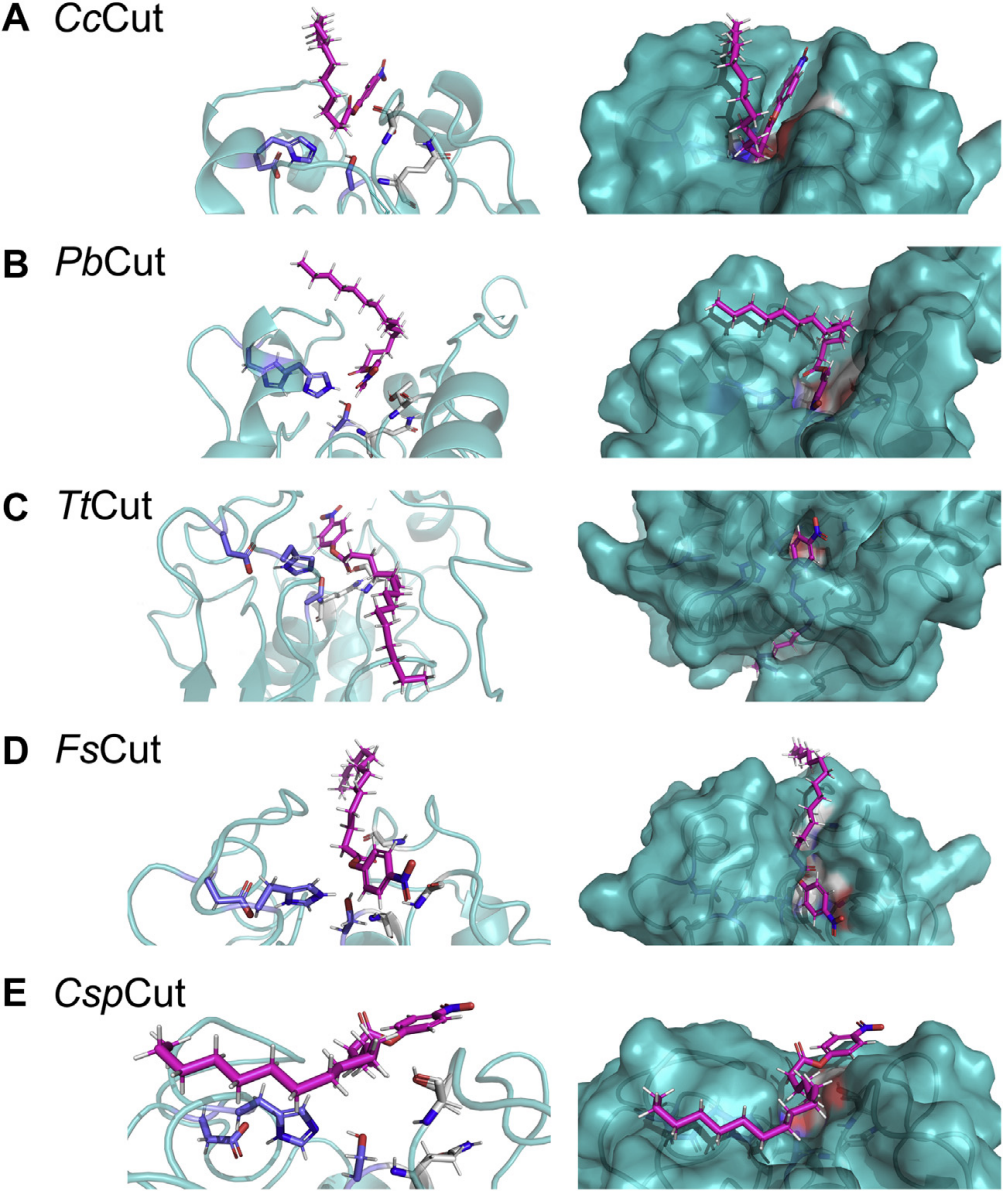
**Docking analysis of five cutinases using *p*NP-C**_**16**_**.** Depicted are *Cc*Cut (*A*), *Pb*Cut (*B*), *Tt*Cut (*C*), *Fs*Cut (*D*), and *Csp*Cut (*E*) with the substrate in the most stable conformation. Enzyme backbones and surfaces are depicted in teal, ligands in *magenta*, catalytic triads in *dark blue*, and oxyanion holes in *gray*. In combination with our phylogenetic analysis, these docking results suggest three distinct modes of enzyme–substrate interaction. *Tt*Cut (*C*) and *Fs*Cut (*D*) harbor a solvent-exposed catalytic triad, *Cc*Cut (*A*) and *Pb*Cut (*B*) a lipid-like lid, and *Csp*Cut (*E*) has two flexible loops that might undergo a clamshell-like motion for substrate binding. Please note that angles of enzyme display are adjusted to maximize clarity.

To further understand the divergence between the catalytic efficiency and the binding energy with increasing chain lengths, a detailed structure–function analysis was conducted using *p*NP-C_16_ as substrate. Representations of the enzyme surfaces and the active site pockets of the docked structures are summarized in Figure 5. Between the five enzymes, three different pose configurations were acquired, as discussed below.

#### The case of TtCut and FsCut—cutinases with a solvent-exposed active site

The fits of the ligand in the active site pocket were very good for *Tt*Cut and *Fs*Cut, as assessed by docking score (Table S2), proximity of the carbonyl carbon to the catalytic serine (2.9–3.2 Å), and the close distance between the carbonyl oxygen and the oxyanion hole to allow for h-bond formation (2.1–3.6 Å). This enzyme–ligand interaction corresponds well with studies conducted on *F. solani* (37), *A. oryzae* (23), and *M. cinnamomea* (14) crystal structures, as well as with the *Alternaria brassicicola* cutinase CUTAB1 model (17). These four cutinases are also located in the same part of the phylogenetic tree (Fig. 1, clades A, B, and C; Fig. S2), indicating their relative similarity in sequence. Our docking results, hence, suggest that the structure–function relationship of *Tt*Cut and *Fs*Cut, as well as other related cutinases located in clades A, B, and C of the phylogenetic tree, follows the traditional description of the cutinase enzyme family, involving a solvent-exposed active site that can enter a catalytic cycle without the need for further loop rearrangement.

#### The case of CcCut and PbCut—cutinases with a lipase-like lid

Docking of *Cc*Cut and *Pb*Cut with *p*NP-C_16_ resulted in a bent configuration of the ligand (Fig. 5). Although the proximity of the carbonyl carbon to the catalytic serine (3.5–4.2 Å) is acceptable, this bent configuration is clearly different from the other cutinases, in which the *p*NP-C_16_ goes straight over the enzymes' binding face (Fig. 5). In case of *Pb*Cut it was further not possible to achieve a pose with a h-bond to either amino acid residue of the oxyanion hole (Thr43 or Gln119, Table 2), independent of the docking restraints applied. As this is an essential part for enzyme–substrate interaction (37), this pose would not lead to completion of the catalytic cycle. Docking of *p*NP-C_16_ with *Cc*Cut resulted in h-bond formation with the oxyanion residue Thr42.

Interestingly, both *Cc*Cut and *Pb*Cut have the *T. reesei* cutinase structure as template. *Cc*Cut in the enzyme's native form (PDB: 4PSC), where the lid is closed, and *Pb*Cut in its complexed from with the C11Y4 phosphonate inhibitor (PDB: 4PSE), where the lid is swung out. The configurational change of the lid described for the *T. reesei* cutinase structures has led to the hypothesis that this cutinase is functioning similar to lipases, *i.e.*, with a significant rearrangement of the loop upon substrate contact (33). Indeed, the overall structure of *Pb*Cut, with one loop swung out from the main enzyme body (Fig. S3*C*), closely resembles that of the “open” *T. reesei* cutinase structure (33). The phylogenetic closeness of both *Cc*Cut and *Pb*Cut with the *T. reesei* cutinase (located in or close to clade F, Fig. S2) further supports the notion that these three cutinases share properties with each other. Although the role of the moving loop cannot be investigated further with the docking analysis used herein, our results support the hypothesis that this lid movement, similar to lipases, is required for some cutinases to successfully bind bulky ligands such as *p*NP-C_16_. As *Pb*Cut showed good catalytic efficiency against the long chain *p*NP-substrates (Table 2), further investigation of this type of cutinase will be important to further understand and exploit its ability to act on bulky substrates.

#### The case of CspCut—a cutinase with flexible loops flanking the active site that might undergo clamshell-like motion

**Figure 6:**
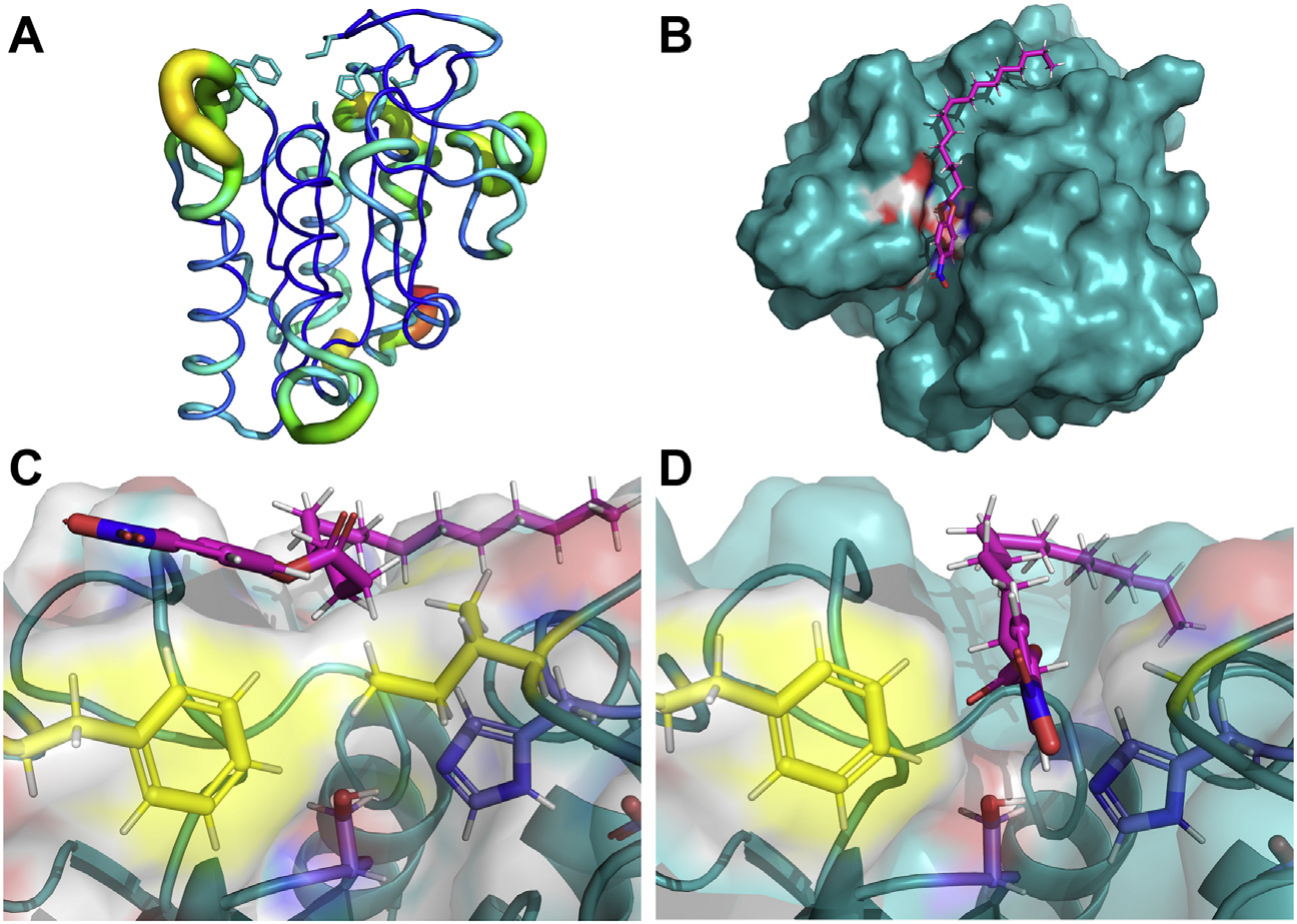
**The role of the “gatekeeper” amino acids Phe52 and Ile176 on enzyme–ligand interaction of *Csp*Cut.***Panel A* depicts the b factor (atomic displacement parameter), where increased mobility is indicated by a broadening of the backbone cartoon and a color scale going from *dark blue* (little mobility) to *red* (highest mobility). The docked surface structure of the *in silico* Gly176Leu variant with *p*NP-C_16_ is shown in *panel B*. Panels *C* and *D* illustrate the active site of the wild-type (*C*) and Gly176Leu variant (*D*) enzyme, where the catalytic triad is marked in *blue*, the “gatekeeper” amino acid residues in *yellow*, and the ligand in *magenta*. Please note, chain B of the crystal structure 2CZQ was used for docking analysis.

In case of *Csp*Cut, it was not possible to gain a realistic pose under any of the applied docking restraints, with the *p*NP-C_16_ too far away from the catalytic serine (>6 Å) and the carbonyl oxygen pointing away from the active site toward the solvent. Because *Csp*Cut has a resolved crystal structure (PDB: 2CZQ), a closer structural examination was possible. Similar to cutinases from other organisms (14, 17, 23, 34, 37), the width of the active site cleft in *Csp*Cut is restricted on top by two “gatekeeper” amino acid residues, here Phe52 and Ile176 (Fig. 6).

These “gatekeeper” residues, *e.g.*, Leu81 and Val184 in *Fs*Cut (Fig. S3), have been suggested to partake in substrate recognition and binding (37). Due to the closeness of these residues on opposite loops, they also have been suggested to restrict binding of bulkier ligands. Hence, engineering studies on *F. solani* (34, 37) and *A. brassicicola* (17) cutinases have replaced these amino acid residues (Leu, Ile, Val, Phe; Fig. S3) with shorter ones, resulting in enhanced activity toward larger molecules. Following these findings, we created an *in-silico* Ile176Gly variant. The achieved pose of the substrate molecule was closer to what could be expected (Fig. 6), with improved proximity of the carbonyl carbon to the catalytic serine (3.6 Å) and a h-bond formed between the carbonyl oxygen and oxyanion hole residue Thr17 (2.4 Å). The docking score also drastically improved (Table S2). However, *Csp*Cut already has superior affinity and excellent catalytic efficiency against *p*NP-C_8_ to *p*NP-C_16_ (Table 3), suggesting that enzyme engineering to improve catalysis for this kind of substrate is not necessary. Rather, we hypothesize that high flexibility of the two loops bracketing the active site, as reported for *F. solani* (37) and *M. cinnamomea* (14) cutinases, might allow for enough movement to enclose the ligand in the active site. Indeed, the b factor of the crystal structure shows high flexibility of the loop harboring the oxyanion hole and to a lesser extent also the one containing the catalytic triad (Fig. 6). Hence, it might be possible that *Csp*Cut undergoes a clam-shell-like motion, where it opens up to allow the ligand to enter the active site and then closes until the catalytic cycle is completed and the product released. Comparison of the two proteins contained in the 2CZQ crystal structure further supports this hypothesis. Hence, Phe52 on chain B (used herein for docking, due to several conflicts in amino acid positions in the chain A protein) is found on a loop facing the binding cleft (as shown in Fig. 6), whereas in chain A Phe52 is instead facing away from the binding cleft as the loop is found swung away from the active site. However, further testing of this hypothesis could not be achieved with the simple docking analysis used in this study and will remain a target for future studies.

## Conclusion

Here we investigated the biodiversity among CE5 cutinases to gain knowledge on enzyme kinetics and structure–function relationships, which can help accelerate use of this poorly studied enzyme family. Our phylogenetic analysis of CE5 cutinases and characterization of phylogenetically diverse cutinases significantly adds to the current knowledge on this enzyme family's properties. Results show that cutinases that have been structurally investigated to date are closely grouped in the family's phylogenetic tree. Characterization against *p*NP-substrates with consecutively longer alkyl chains further demonstrated that each of the nine enzymes has an individual activity fingerprint. Interestingly, sequences of the five cutinases with the highest activity against *p*NP-C_12_ and C_16_ were scattered across the phylogenetic tree, suggesting that no single feature can be linked to high activity against bulky and hydrophobic substrates. We suggest that a combination of characteristics gives raise to this desirable trait, including the configuration of the binding cleft harboring the catalytic triad, extent, and hydrophobicity of the loops bracketing the binding cleft, as well as the length of “gatekeeper” residues enclosing the binding cleft. Detailed structure–function analysis of the five sequence-diverse cutinases further suggests that different clades of the phylogenetic tree harbor enzymes with different modes of substrate interaction. These include the traditionally described cutinase with the active site exposed to the solvent (as observed for *Fs*Cut and *Tt*Cut), substrate interaction over a lipase-like lid (*Cc*Cut and *Pb*Cut), and substrate binding involving a clamshell-like movement of likely flexible loops bracketing the binding cleft (*Csp*Cut). Kinetically, however, these enzymes share one trait. Although turnover rates are decreasing with increasing *p*NP-substrate length, the substrate affinity (*i.e.*, *K*_*M*_ and binding energies) is improving significantly. Our study shows that we only started to understand the diversity of cutinases and that there is a large knowledge gap to fill. To exploit this interesting enzyme class, further studies exploring the structure–function diversity, including resolving more cutinase structures from diverse sequences, are required.

## Experimental procedures

### Phylogenic analysis

CE5 sequences to be analyzed were chosen according the decision tree and description shown in the supporting information (Fig. S1). The selected amino acid sequences were manually trimmed to include only the catalytic domain and subsequently aligned using MUSCLE (38). The phylogenetic tree was constructed using IQ-TREE (39), with the default settings, including automatic determination of the best substitution model (WAG+F + I + G4) and 1000 ultrafast bootstraps. The tree was visualized using iTOL (40).

### Cloning of cutinase genes into *P. pastoris*

Nine genes were selected for cloning into *P. pastoris* and kinetic characterization against *p*NP-substrates. Source organisms, accession numbers, and references can be found in Table 1. Genes were ordered on high-copy *Escherichia coli* plasmids *via* Twist Bioscience, where an N-terminal 6xHis-Tag was added, and the sequence codon optimized for *P. pastoris*, using the company's own software. Amplification of the cutinase Twist plasmids and the *Pichia* integration plasmid pPICZα A (Invitrogen) was performed in *E. coli* BL21, where cells were made chemically competent prior to transformation by heat shock. Plasmids were purified using the GeneJET Plasmid Miniprep Kit (Thermo Scientific) and subjected to a restriction digest using EcoRI and NotI (Thermo Scientific) at 37 °C for 1 h. After inactivation at 65 °C for 5 min, ligation was performed without further DNA fragment clean-up at a volumetric pPICZα to cutinase gene ratio of 1, using T4 ligase (Thermo Scientific) at 4 °C overnight. Without further cleanup, *E. coli* BL21 was transformed with the ligation mix, containing the cutinase-pPICZα plasmids, using zeocin as selection marker. Correct plasmid configurations were tested by colony PCR using the primers AOXfw (5′→3′ TAATTATTCGAAACGAT GAGATTTCCTTCAATTTTTACTGC) and AOXrev (5′→3′ CTGACATCCTCTTGATTAGAATCTAGCAAGAC), binding to the AOX promoter and terminator region, respectively. After amplification and purification using the GeneJET kit, the cutinase-pPICZα plasmids were linearized by SacI (Thermo Scientific) and then transformed into *P. pastoris* SMD1168H (Invitrogen) using the LiCl method and screening for zeocin resistance. Cutinase production was tested by a microscreening in 96-deep well plates with a 1 ml well volume. For this, 600 μl of YPGly10-Zeo100 (yeast extract 10 g l^−1^, peptone 20 g l^−1^, glycerol 10 g l^−1^, zeocin 100 μg l^−1^) was inoculated with a single colony, and the plate incubated at 30 °C and 500 rpm for 24 h in an incubator shaker (ThermoMixer C, Eppendorf). Hundred microliters was then transferred to a new plate with 500 μl of fresh YPGly-Zeo100. After incubation for 24 h (30 °C, 500 rpm), gene expression was induced by adding four times 6 μl of methanol every 12 h. Cutinase expression was verified by SDS-PAGE and activity screening using *p*NP-C_4_ as described below. No *p*NP activity was detected in the supernatant of the *P. pastoris* SMD1168H parental strain (not harboring a cutinase gene). For each of the nine cutinase-harboring strains 10 to 20 clones were screened. SDS-PAGE results of the successful clones can be found in Figure S5.

### *P. pastoris* cultivation for cutinase production

Recombinant *Pichia* strains were kept in glycerol (30% v/v) stocks at −80 °C. Prior to cultivations they were streaked on YPD-Zeo100 plates (yeast extract 10 g l^−1^, peptone 20 g l^−1^, glucose 20 g l^−1^, zeocin 100 μg l^−1^) and incubated for 72 h at 30 °C. One colony of each cutinase was then used to inoculate a 2 ml YPGly10-Zeo100 overnight culture in 10 ml culture tubes, which were incubated in an orbital incubator shaker at 150 rpm and 30 °C. In total, 0.5 ml of the overnight culture was then used to inoculate 50 ml of YPGly20-Zeo100 (yeast extract 10 g l^−1^, peptone 20 g l^−1^, glycerol 20 g l^−1^, zeocin 100 μg l^−1^) in 300 ml baffled shake flasks closed with two layers of miracloth (Merck). Incubation was at 30 °C, 200 rpm in an orbital incubator shaker for 48 h. Afterward, the temperature was lowered to 28 °C, and protein production was induced by adding 500 μl of pure methanol every 12 h for 48 h (2 ml total addition). After an additional incubation for 48 h, the supernatant was harvested by centrifugation. The supernatant was concentrated ten-fold (Macrosep Advance, 10 kDa cutoff, Pall Laboratories) and buffer exchanged into NaPh7 (50 mM sodium phosphate buffer, pH 7) for initial screening of cutinase activity or into binding buffer (20 mM Tris-HCl buffer, pH 8) for protein purification.

### Protein purification

Protein purification by affinity chromatography was accomplished using nickel beads (Ni Sepharose Excel, GE healthcare), which were primed for use by washing five times with and incubating for 10 min in binding buffer. Then the supernatant, concentrated and buffer exchanged into binding buffer, was mixed with the primed beads and incubated for 1 h at 4 °C under constant stirring. Afterward, the beads were packed into self-pack column, and the flow-through was discarded. The beads were washed twice with three column volumes of washing buffer (20 mM Tris-HCl with 25 mM NaCl, pH8) and bound protein eluted with two times three column volumes of elution buffer (20 mM Tris-HCl with 25 mM NaCl and 250 mM imidazole, pH7), followed by immediate buffer exchange (Macrosep Advance, 10 kDa cut off) into NaPh7. SDS-PAGE was used to verify successful enzyme purification and enzyme purity (gel pictures can be found in the supporting information; Fig. S5). Enzymes were stored at 4 °C until activity analysis conducted within 72 h after purification, during which no activity loss was observed. Protein concentrations were determined by Abs_280_ and the calculated extinction coefficients (41).

### Activity measurement using *p*NP-substrates

Cutinase activity was evaluated against *p*NP-substrates with consecutively longer fatty acid chains. All reactions were performed in 96-well plates (Greiner Bio-One 675801) with 200 μl total volume. Substrate solutions of *p*NP-C_2_, *p*NP-C_4_, *p*NP-C_8_, *p*NP-C_12_, and *p*NP-C_16_ were prepared in a concentration range of 0.5 to 250 mM in absolute ethanol and stored at −20 °C. In principle, all reactions were performed with the following protocol. For the substrates *p*NP-C_2_, *p*NP-C_4_, *p*NP-C_8_, and *p*NP-C_12_, 140 μl of NaPh7 was mixed with 50 μl of enzyme solution and preheated to 40 °C. Reactions were started by adding 10 μl of substrate solution. Due to reduced solubility, *p*NP-C_16_ solutions and NaPh7 were preheated to 70 °C and then mixed at ratio 1:15 before cooling it down to 40 °C. In total, 150 μl of this solution was then mixed with 50 μl of enzyme to start the reaction. The release of *p*NP over time was followed at 405 nm using a SpectroStar Nano (BMG LABTECH) plate reader, and absorbance quantified against a *p*NP standard curve. *p*NP released by autohydrolysis was subtracted by running one control per each enzyme reaction, replacing the enzyme with buffer. Activities were derived from the linear area of the progress curve.

The initial activity screening of all nine cutinases was performed in duplicate experiments in the concentrated and buffer-exchanged cultivation supernatant (protein concentration range in the reaction 0.004–0.4 mg ml^−1^) using 62.5 mM (*p*NP-C_2_, *p*NP-C_4_, *p*NP-C_8_) and 31.25 mM (*p*NP-C_12_, *p*NP-C_16_) as substrate in NaPh7 buffer.

For in-depth kinetic characterization, the purified enzymes were used (protein concentration range in the reaction 0.1–4 μM). First, pH dependency profiles were established between pH 4 and 9, using 62.5 mM *p*NP-C_8_ as substrate and 50 mM sodium phosphate buffer (pH 4–7) and 50 mM Tris-HCl buffer (pH 8–9). All cutinases had a discrete maximum at pH 7 (Fig. S6), so NaPh7 was used as buffer. For determination of kinetic parameters, the specific activities (1 μmol of *p*NP released per minute and mg enzyme), measured for increasing substrate concentrations until saturation is reached, were plotted against the respective substrate concentration and fitted by the nonlinear MM equation using GraphPad Prism. For each kinetic characterization, 6 to 16 substrate concentrations were measured as independent experiments. The entire data set was then repeated, and data depicted herein represent mean values and the data spread.

### Docking simulations

Docking simulations were performed using the flexible Glide Standard Precision (SP) function of the Maestro 12.3 program (Schrödinger, Inc). Substrate molecules were prepared using ChemDraw (PerkinElmer). Crystal structures of the *Csp*Cut (PDB: 2CZQ; (42)) and *Fs*Cut (PDB: 3QPC; (43)) were used. For *Cc*Cut, *Tt*Cut, and *Pb*Cut, enzyme models were established using SWISS MODEL (https://swissmodel.expasy.org/), iTASSER (https://zhanglab.ccmb.med.umich.edu/I-TASSER/), and Phyre2 (http://www.sbg.bio.ic.ac.uk/∼phyre2/html/page.cgi?id=index) algorithm. Models were chosen after careful examination and comparison of the models with their templates using Pymol (Schrödinger), where focus was set on alignment of the catalytic triad and the oxyanion hole, as well as successful closure of predicted disulfide bonds. Models, crystal structures, and substrate molecules were loaded into the Maestro program and adjusted to the experimental pH of 7 using the EPIK module. A receptor grid box with 20 Å axes lengths, centered at the catalytic serine, was generated. It contained information for two docking constraints: a spatial constraint (nuclear Overhauser effect; NOE) to bring the ester bond into proximity to the catalytic serine; and a hydrogen bond constraint (hbond) to orient the carbonyl oxygen of the ester bond toward the oxyanion hole. For ligand docking, hydrogen bonds were rewarded, and an EPIK state penalty added to the docking score. Each enzyme was docked with each substrate under four constraints: using no constraints, NOE, hbond, or both. Poses were evaluated by the user according to the proximity of the ester oxygen bridge to the catalytic serine, the orientation of the carbonyl oxygen to the oxyanion hole, as well as the docking and glide rmodel score. For the best pose, the binding energy was then calculated using the Prime MM-GBSA function in Maestro (29). The constraints of each pose are summarized in Table S2. Overlays and enzyme structures presented herein were prepared using Pymol 4.6 (Schrödinger).

## Data availability

All data of the presented study is included in the paper's main body or in the supporting information.

## Supporting information

This article contains supporting information.

## Conflict of interest

The authors declare that they have no conflicts of interest with the contents of this article.
