# Supplementary material for: Phylogenetic analysis and in-depth characterization of functionally and structurally diverse CE5 cutinases
Source: J Biol Chem. 2021 Oct 13;297(5):101302. doi: 10.1016/j.jbc.2021.101302 (PMC8577158; doi:10.1016/j.jbc.2021.101302)
Supplement: Figures S1–S6 and Tables S1 and S2 [file mmc1.pdf]

Supplementary Information SI-1

Phylogenetic analysis and in-depth characterization of functionally and structurally diverse CE5 cutinases

Vera Novy[1,2], Leonor Vieira Carneiro[1], Jae Ho Shin[1], Johan Larsbrink[1,2], Lisbeth Olsson[1,2]

[1]Department of Biology and Biological Engineering, Division of Industrial Biotechnology, Chalmers University of Technology, SE-412 96 Gothenburg, Sweden

[2]Wallenberg Wood Science Center, Chalmers University of Technology, Gothenburg, Sweden

SI-1 contains:

Supplementary Figures: S1 to S6

Supplementary Tables: S1 to S2

Supplementary Figure S1

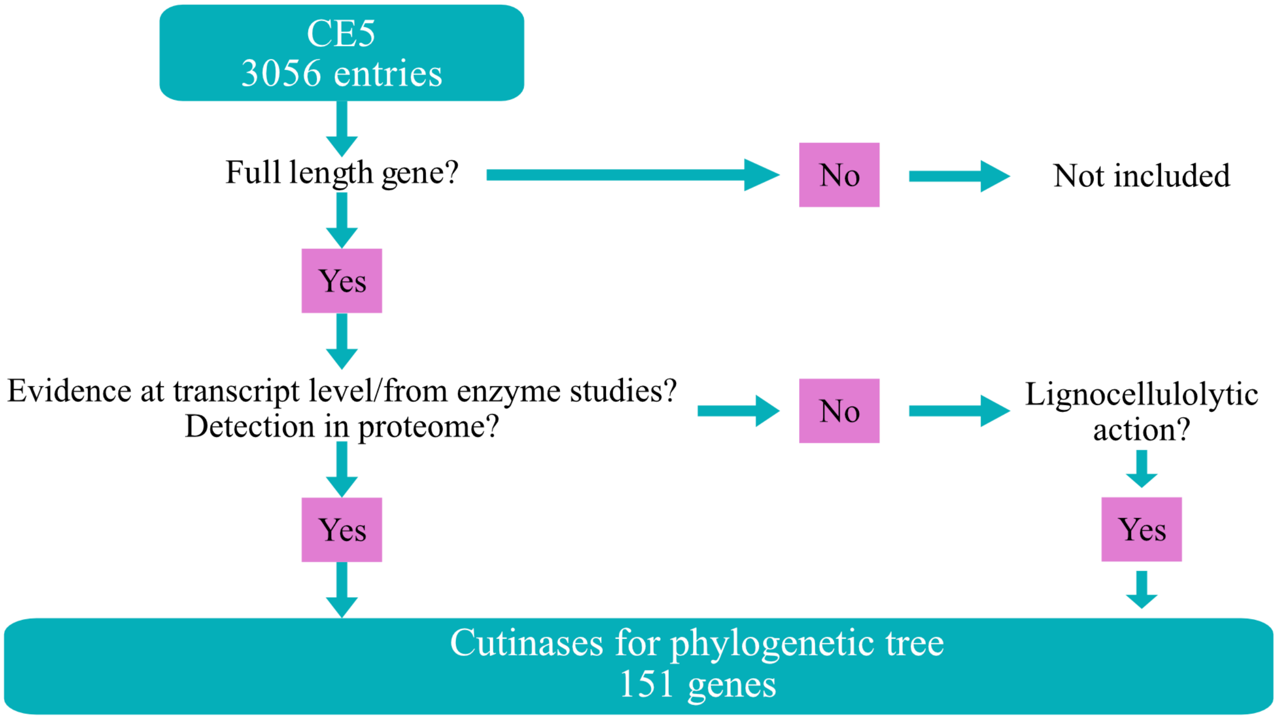

Figure S1: Decision tree to condense all 3056 CE5 entries (www.cazy.org; time frame of data mining: February through May 2020; updated January 2021) to the 151 genes presented in the phylogenetic tree in Figure 1. In the first step, gene fragments were eliminated. In the second step, it was checked if the entry of a full length gene has experimental evidence at transcript level, if it was detected in the proteome, or if studies on the recombinant enzyme are available (as opposed to being annotated only). If yes, those genes were included for phylogenetic analysis. If not, further investigation was conducted to analyze if the gene or host organism can be linked to lignocellulolytic action (e.g. lignocellulolytic fungi, plant pathogens). If so, that gene was additionally included. From all 151 genes, we then collected the amino acid sequence and conducted the phylogenetic analysis, as described in the main body of this paper. A list of all 151 enzymes and their accession numbers is provided with this paper (Supplementary Information SI-2).

Supplementary Figure S2-1

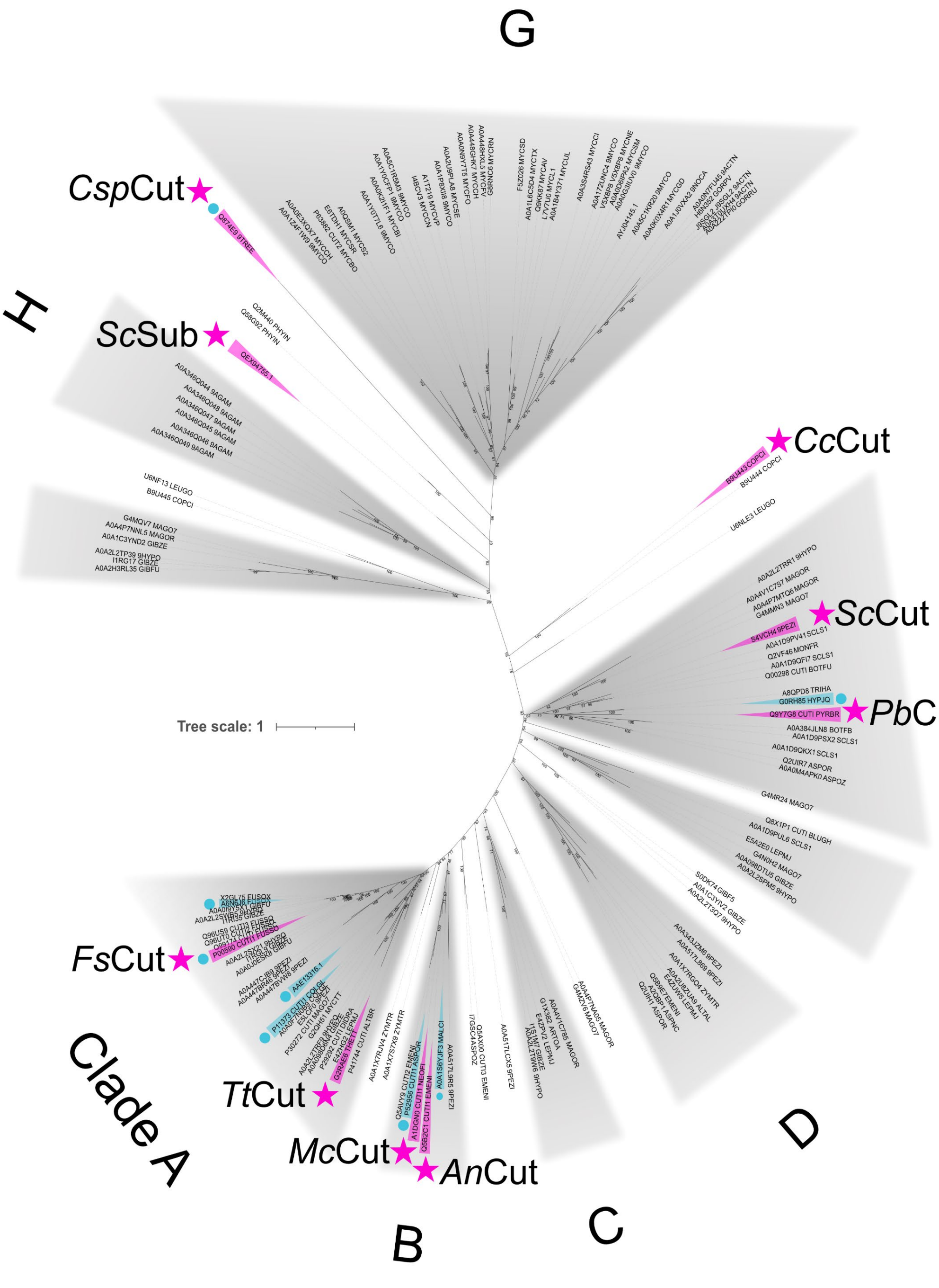

Supplementary Figure S2-2

**Division**	**Species**	**Count in respective clade**								
		A	B	C	D	E	F	G	H	I
Ascomycetes	*Alternia sp**	1			**2**					
	Arthrobotrys sp			**1**						
	*Aspergillus sp**		**3**		**3**		**2**			
	*Blumeria sp**					**1**				
	*Botryotinia sp**						**2**			
	*Ceratobasidium sp**								**6**	
	*Didymella sp**	2								
	*Fusarium sp**	14		**2**		**2**	**1**			**4**
	*Glomorella sp**	3								
	Humicola sp	1								
	*Leptosphaeria sp**	1		**1**	**1**	**1**				
	Malbranchea sp		**1**							
	*Monilinia sp**						**1**			
	Neofusicoccum sp				**1**					
	Podospora sp	3								
	*Pyrenopeziza sp**						**1**			
	*Pyricularia sp**	1		**1**		**1**	**3**			**2**
	*Sclerotonia sp**					**1**	**4**			
	*Sirococcus sp**						**1**			
	Thielavia sp	1								
	Trichoderma sp						**3**			
	*Venturia sp**		**1**		**1**					
	*Zymoseptoria sp**				**1**					
Actinobacteria	*Mycolibacterium sp*							**10**		
	Nocardia sp							**1**		
	Rhodococcus sp							**5**		

Figure S2: The main clades of the phylogenetic tree (Figure S2-1) and the main genera and divisions (Figure S2-2) found in them. Families marked with an asterisk are plant pathogens. A list of all enzymes and their corresponding clade is provided with this paper (SI-2).

Supplementary Figure S3-1

A) *Cc*Cut

SWISS-MODEL
Main Template PDB: 4PSC

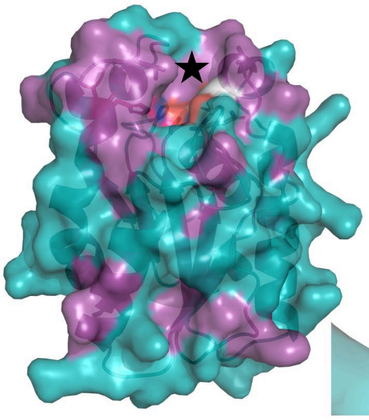

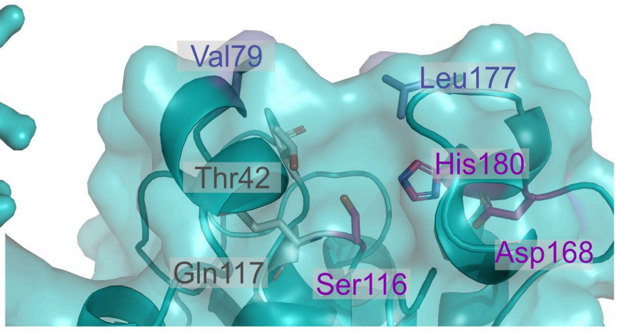

B) *Sc*Sub

Phyre2
Main Template PDB: 4PSC

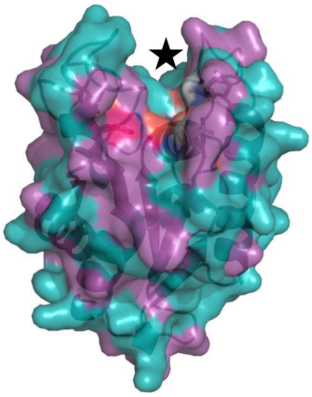

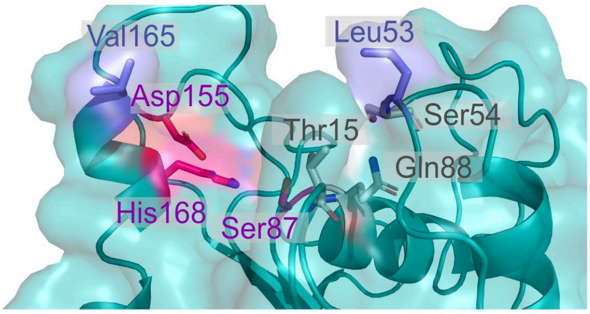

C) *An*Cut

Phyre2
Main Template PDB: 5X88

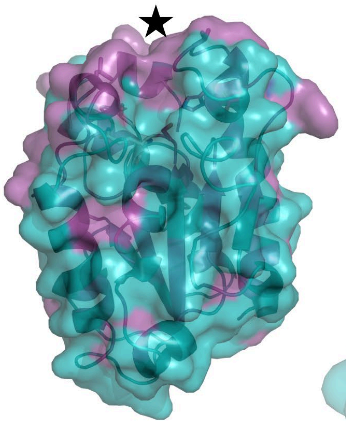

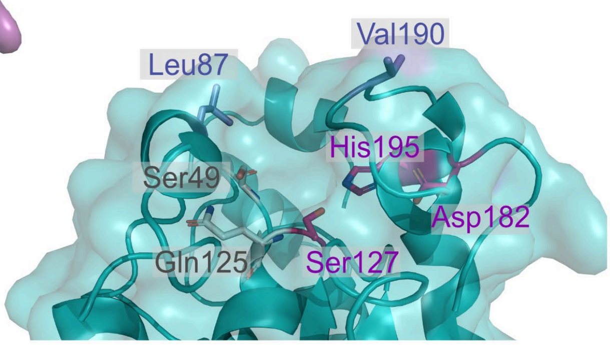

C) *Pb*Cut

SWISS-MODEL
Main Template PDB: 4PSE

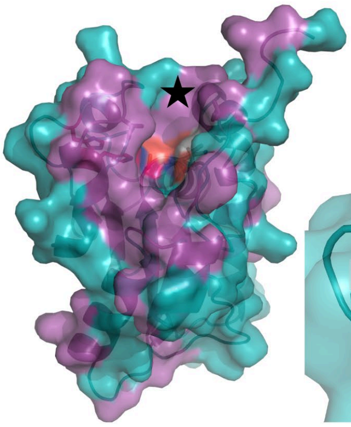

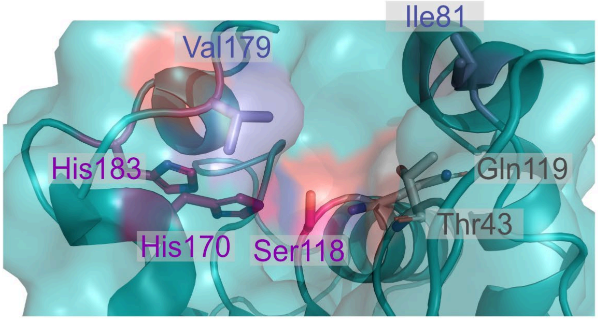

Supplementary Figure S3-2

E) *Sc*Cut

SWISS-MODEL

Main Template PDB: 4PSD

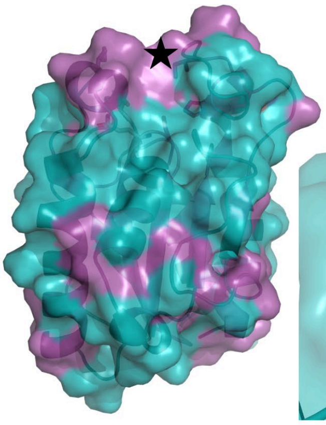

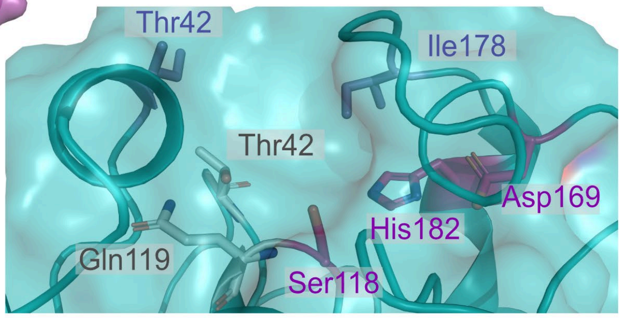

F) *Mc*Cut

SWISS-MODEL

Main Template PDB: 3GBS

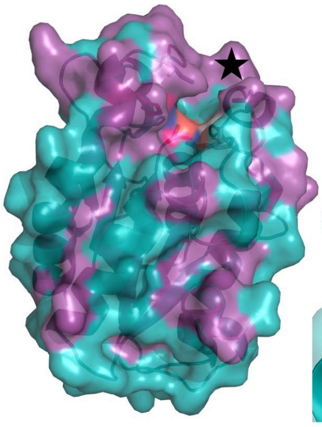

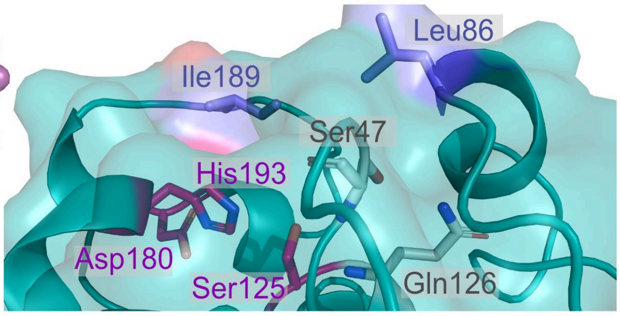

G) *Tt*Cut

Phyre2

Main Template PDB: 3DD5

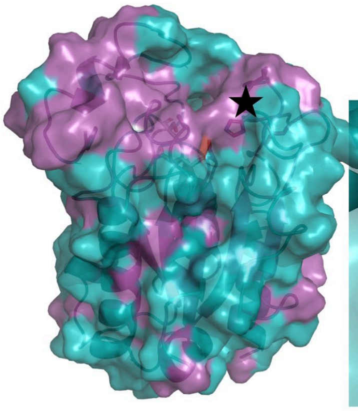

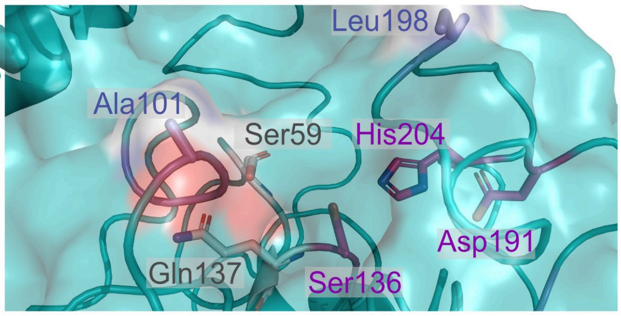

H) *Csp*Cut

Structure PDB: 2CZQ

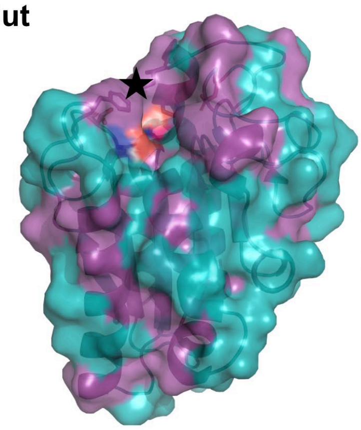

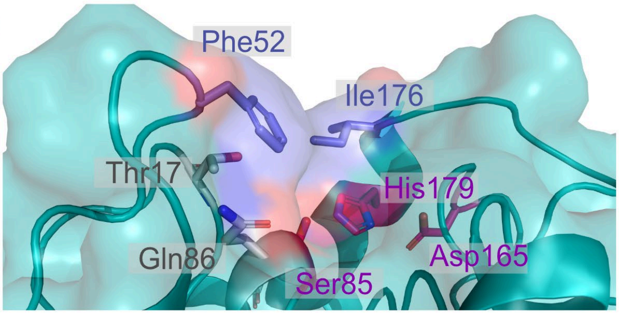

Supplementary Figure S3– 3

F) *Fs*Cut

Structure PDB: 3QPC

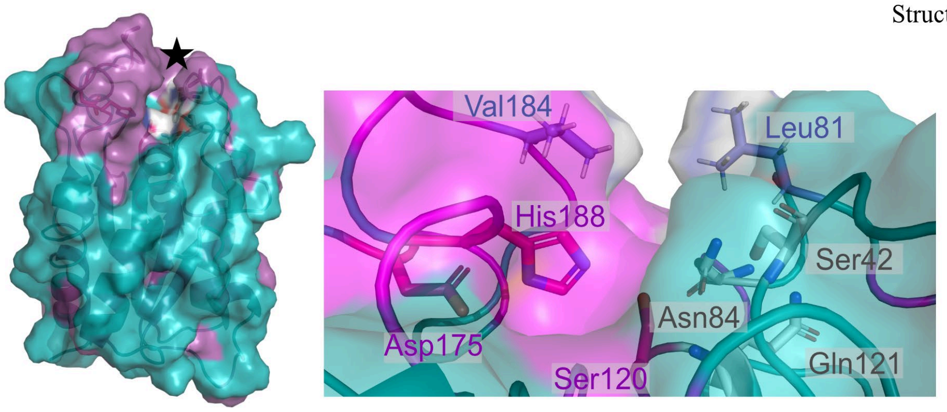

Figure S3: Models of enzyme surfaces and active sites of the nine selected cutinase enzymes. Surfaces are shown in teal, with hydrophobic residues highlighted in magenta. The binding cleft containing the active site is marked with a star. The amino acid residues of the catalytic triad are marked in dark magenta, the oxyanion hole in grey, and those considered to be involved in substrate recognition in blue. Figure panels further include which algorithm gave the best model and indicate the PDB accession number of the prime template. Please note that angles of enzyme structures were adjusted for clarity.

Figure S4

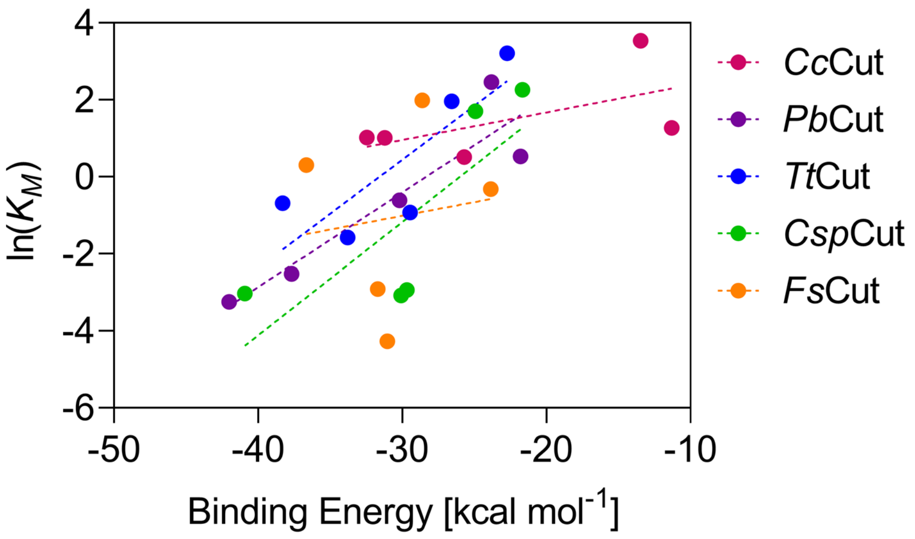

Figure S4: Correlation between binding energy and K_M in *Cc*Cut, *Pb*Cut, *Tt*Cut, CspCut, and *Fs*Cut against the five *p*NP substrates. The calculated binding energies (from *in-silico* docking experiments) and K_M values are summarized in Table 2. Because of the observed exponential behavior, a semi-logarithmic depiction was chosen. The dashed lines show the linear regression.

Supplementary Figure S5

A)

B)

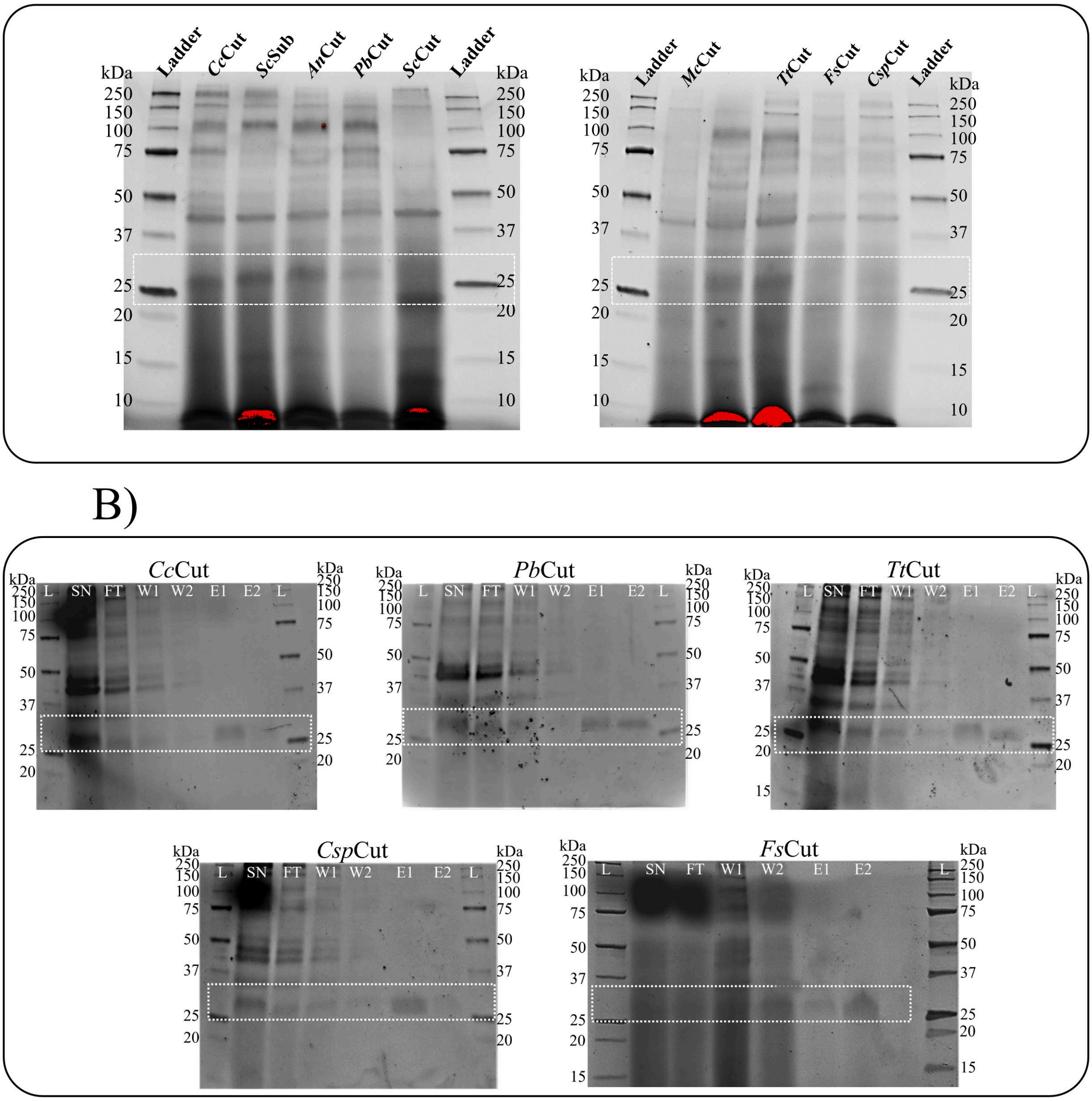

Figure S5: (A) SDS-PAGE of all positive cutinase clones used for further characterization. Depicted is the supernatant from the micro screening as described in the method section. The cutinases are found around the 25 kDa range, as indicated in blue. (B) Purification of five cutinases used for in-depth characterization. Depicted are the following lanes: Ladder (“L”); cultivation supernatant (“SN”); flow through after applying the supernatants to the Ni beads (“FT”); wash fraction 1 (“W1”); wash fraction 2 (“W2”); elution fraction 1 (“E1”); and elution fraction 2 (“E”). Please note, the large band on the top of each gel is the unconsumed methanol used for induction of protein expression.

Supplementary Figure S6

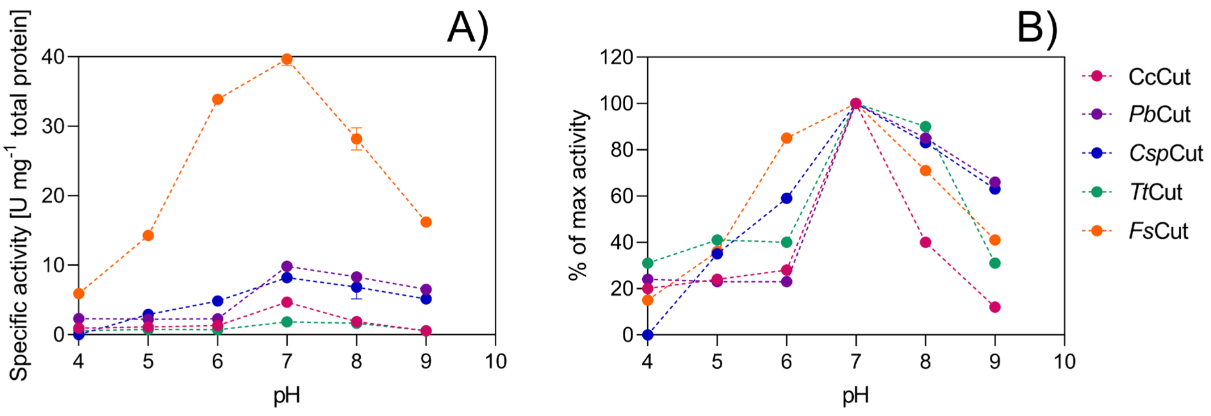

Figure S6: pH profiles (pH 4 to 9) of the five cutinases selected for further kinetic characterization. Depicted are the specific activities (given in U mg^{-1} total protein in the cultivation supernatant) at pH 4-9 (panel A) as well as the relative distribution of activities for reasons of clarity (panel B). Activities were measured against 3.13 mM *p*NP-C8 substrate in 50 mM sodium citrate (pH 4-6) and 50 mM sodium phosphate (pH 7-9) buffer. Data points represent average values of duplicate experiments and panel A additionally contains error bars with the spread.

Table S1

Table S1: Specific activity of 9 cutinases measured against the *p*NP-substrates in the concentrated supernatant of *P. pastoris* cultivation[1)].

	***p*NP-C$_2$**	***p*NP-C$_4$**	***p*NP-C$_8$**	***p*NP-C$_{12}$**	***p*NP-C$_{16}$**
**Cutinase**	**Specific Activity [U $mg_{Total\ Protein}^{-1}$]**				
***Cc*Cut**	0.3 ± 0.0	0.9 ± 0.0	0.4 ± 0.1	0.7 ± 0.1	0.1 ± 0.0
***Sc*Sub**	5.1 ± 0.1	13.7 ± 0.5	1.6 ± 0.1	0.9 ± 0.2	0.2 ± 0.0
***An*Cut**	0.3 ± 0.0	1.0 ± 0.1	2.2 ± 0.2	0.1 ± 0.0	0.1 ± 0.0
***Pb*Cut**	3.2 ± 0.1	2.5 ± 0.0	1.0 ± 0.0	1.1 ± 0.0	0.5 ± 0.0
***Sc*Cut**	40.4 ± 3.6	153.4 ± 17.0	27.3 ± 1.2	17.1 ± 1.1	0.5 ± 0.0
***Mc*Cut**	5.9 ± 0.4	26.9 ± 0.7	2.1 ± 0.3	0.5 ± 0.1	0.3 ± 0.0
***Tt*Cut**	0.1 ± 0.0	0.1 ± 0.0	0.4 ± 0.0	0.3 ± 0.0	0.0 ± 0.0
***Fs*Cut**	142.5 ± 8.8	229.5 ± 3.9	107.1 ± 3.4	8.2 ± 0.8	3.0 ± 0.2
***Csp*Cut**	19.1 ± 0.2	50.8 ± 1.4	36.7 ± 1.7	25.8 ± 0.7	11.5 ± 0.4

1) Data represent mean values and the spread of duplicate experiments.

Table S2

Figure S2: Constraints applied, docking score, and glide emodel of docking experiments. Binding energies are depicted in Table 2.

<table>
<tr><th></th><th>CcCut</th><th>PbCut</th><th>TtCut</th><th>CspCut</th><th>FsCut</th></tr>
<tr><td colspan="6">Docking score</td></tr>
<tr><td>pNP-C_2</td><td>-2.7</td><td>-4.5</td><td>-2.9</td><td>0.3 (-2.2)</td><td>-0.1</td></tr>
<tr><td>pNP-C_4</td><td>2.4</td><td>-2.7</td><td>-2.0</td><td>1.9 (-2.7)</td><td>-3.4</td></tr>
<tr><td>pNP-C_8</td><td>-1.1</td><td>-0.3</td><td>-0.9</td><td>1.0 (0.1)</td><td>-0.5</td></tr>
<tr><td>pNP-C_{12}</td><td>-2.1</td><td>-2.0</td><td>-3.7</td><td>6.5 (-0.8)</td><td>-2.4</td></tr>
<tr><td>pNP-C_{16}</td><td>-1.3</td><td>-2.0</td><td>-3.3</td><td>-0.5 (-0.9)</td><td>-0.3</td></tr>
<tr><td colspan="6">Glide emodel</td></tr>
<tr><td>pNP-C_2</td><td>-1.9</td><td>-27.1</td><td>1.8</td><td>-0.7 (-13.3)</td><td>-3.6</td></tr>
<tr><td>pNP-C_4</td><td>16.4</td><td>-21.5</td><td>0.0</td><td>6.9 (-20.6)</td><td>-23.9</td></tr>
<tr><td>pNP-C_8</td><td>-13.3</td><td>-18.8</td><td>-2.6</td><td>0.2 (-17.7)</td><td>-18.9</td></tr>
<tr><td>pNP-C_{12}</td><td>-17.9</td><td>-26.7</td><td>-28.3</td><td>23.9 (-8.1)</td><td>-29.0</td></tr>
<tr><td>pNP-C_{16}</td><td>-8.3</td><td>-29.6</td><td>-26.3</td><td>18.3 (-26.4)</td><td>-21.5</td></tr>
<tr><td colspan="6">Constraints</td></tr>
<tr><td>pNP-C_2</td><td rowspan="5">NOE 4Å;
≥1 hbond</td><td rowspan="4">NOE 4Å;
≥1 hbond</td><td rowspan="3">NOE 3Å;
≥1 hbond</td><td rowspan="3">NOE 5Å;
≥1 hbond</td><td rowspan="5">NOE 3Å;
≥1 hbond</td></tr>
<tr><td>pNP-C_4</td></tr>
<tr><td>pNP-C_8</td></tr>
<tr><td>pNP-C_{12}</td><td>NOE 4Å;
≥1 hbond</td><td rowspan="2">(NOE 4Å);
≥1 hbond</td></tr>
<tr><td>pNP-C_{16}</td><td>NOE 6Å;
≥1 hbond</td><td>NOE 3Å</td></tr>
</table>
